# Integrating Immunotherapy Into Head and Neck Surgery: Bridging Tumor Biology to Perioperative Decision‐Making, a Review

**DOI:** 10.1002/hed.70368

**Published:** 2026-06-22

**Authors:** Nicholas Brian Shannon, Bruce Ashford

**Affiliations:** ^1^ Department of Head and Neck Surgery Illawarra Shoalhaven Local Health District, Wollongong Hospital Wollongong New South Wales Australia; ^2^ Department of Head and Neck Surgery National Cancer Centre Singapore Singapore Singapore; ^3^ Department of Head and Neck Surgery Singapore General Hospital Singapore Singapore; ^4^ Graduate School of Medicine University of Wollongong Wollongong New South Wales Australia

**Keywords:** head and neck squamous cell carcinoma, immunotherapy, neoadjuvant therapy, perioperative, surgical de‐escalation

## Abstract

**Objective:**

To provide a comprehensive review of the biological rationale, clinical evidence, and practical perioperative management of immunotherapy for the head and neck surgeon.

**Summary Background Data:**

Standard treatment for resectable head and neck squamous cell carcinoma (HNSCC) has reached a survival plateau, with over 50% of patients experiencing recurrence. The integration of immune checkpoint inhibitors (ICIs) into the neoadjuvant window represents a paradigm shift toward biologically adapted surgical intervention.

**Results:**

Neoadjuvant immunotherapy capitalizes on an intact immune substrate to create an in situ vaccine, avoiding the post‐surgical immune desert that limits adjuvant efficacy. Emerging phase III data confirm that perioperative ICI significantly improves event‐free survival. Successful implementation requires the surgeon to navigate unique diagnostic challenges, such as distinguishing rare but anatomically risky pseudoprogression from true progression. While combination therapies (chemoimmunotherapy or immunoradiotherapy) yield higher pathologic complete response rates, they also increase toxicity. Intraoperatively, ICI monotherapy generally preserves tissue planes without increasing surgical delays or major wound complications. Standard biomarkers like PD‐L1 and TMB, alongside emerging tools such as liquid biopsy (ctDNA), are essential for patient selection and dynamic monitoring.

**Conclusions:**

The transition to neoadjuvant immunotherapy facilitates future surgical de‐escalation and function‐preserving approaches. To optimize outcomes, the modern surgeon must act as a surgical immunologist, interpreting translational data to guide real‐time operative planning.

## 1 Introduction: Integrating Immuno‐Oncology Into Surgical Practice

The standard of care approach for resectable head and neck squamous cell carcinoma (HNSCC) has traditionally relied on primary surgical resection with risk‐adapted adjuvant therapy. Despite technical refinements in surgical and reconstructive approaches, survival gains remain plateaued. A significant proportion of patients, over 50%, still experience recurrence within 3 years, often incurring substantial morbidity in terms of function and quality of life from aggressive, yet ultimately futile treatments. With approximately 800 000 incident cases annually, this represents a significant global oncology burden.

The integration of immune checkpoint inhibitors (ICI), particularly agents targeting the PD‐1/PD‐L1 axis (e.g., pembrolizumab and nivolumab), has significantly altered this treatment paradigm. While ICI is now standard of care for patients with recurrent or metastatic HNSCC (R/M HNSCC) [1], its success in that setting is tempered by the fact that objective response rates (ORRs) remain modest, approaching only 14% to 22%. This variability reflects the complex nature of HNSCC, a disease characterized by molecular plasticity and dynamic immune evasion [1].

An important clinical shift for the surgical oncologist involves transitioning these agents upstream into the neoadjuvant window [2]. This shifts the focus towards biologically adapted surgical intervention based on real‐time biological response.

While general reviews on immunotherapy exist [1, 3, 4], few address the specific, practical dilemmas facing the operating surgeon. This review translates core T‐cell biology into practical, perioperative decision‐making for the operating surgeon.

## 2 Biological Rationale: From “Immune Desert” to “In Situ Vaccine”

A functional grasp of tumor immunology is now a prerequisite for rational perioperative decision making. The efficacy of neoadjuvant therapeutic sequencing is based on anti‐tumor immunity mechanisms.

### 2.1 T‐Cell Priming and Antigen Recognition

#### 2.1.1 Cancer Immunoediting

**FIGURE 1:**
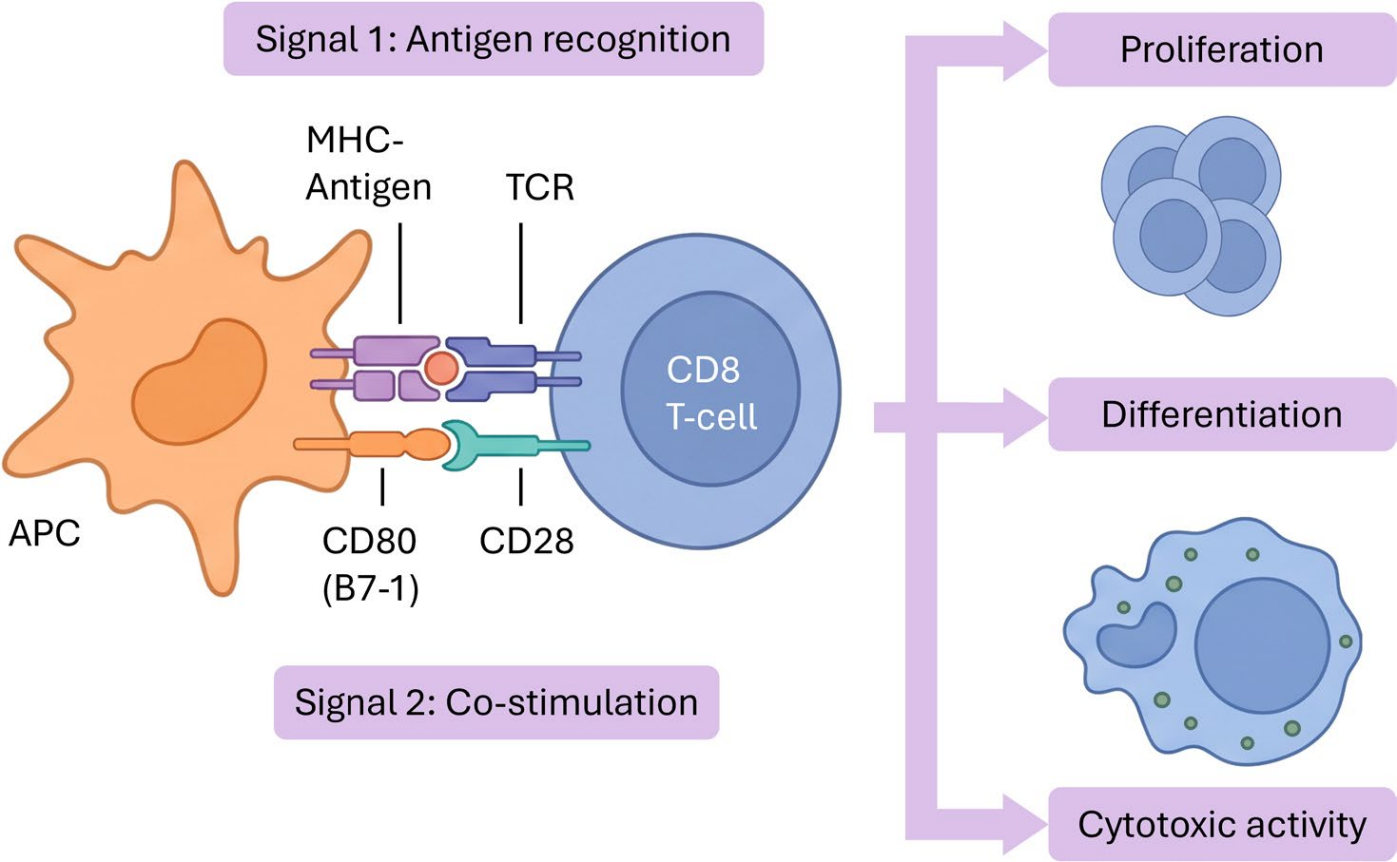
T‐cell activation. The activation of a naïve CD8+ T cell requires a coordinated two‐signal interaction with an antigen‐presenting cell (APC). Signal 1 (Antigen recognition) is mediated by the binding of the T‐cell receptor (TCR) to a specific peptide presented on a Major Histocompatibility Complex (MHC) Class I molecule. Signal 2 (Costimulation) involves the binding of costimulatory ligands on the APC (CD80/B7‐1 or CD86/B7‐2) to CD28 receptors on the T cell. The presence of both signals leads to downstream functional outcomes, including proliferation (clonal expansion), differentiation into effector phenotypes, and the acquisition of cytotoxic activity. [Color figure can be viewed at wileyonlinelibrary.com]

The host‐tumor relationship is defined by “cancer immunoediting”: elimination, where the immune system destroys nascent tumor cells; equilibrium, where surviving cells evolve; and escape, where the tumor evades detection entirely [5]. A robust anti‐tumor response requires the activation of CD8+ cytotoxic T lymphocytes (CTLs) via a coordinated two‐signal system to prevent autoimmunity (Figure 1):

1. *Antigen recognition*: The T‐cell receptor (TCR) binds a specific tumor antigen presented by Human Leukocyte Antigens (HLA) class I molecules.
2. *Costimulation*: A costimulatory receptor on the T‐cell (e.g., CD28) binds to ligands (CD80/CD86) on the antigen‐presenting cell (APC).

The absence of signal leads to T‐cell non‐activation, a state known as anergy.

#### 2.1.2 Antigen Presentation and Cellular Invasion

For antigen recognition, tumors must successfully display antigens on their surface. However, the loss or downregulation of key antigen processing components, observed in up to 70% of HNSCC lesions, is a key mechanism of immune evasion [6]. Beyond presentation loss, HNSCC recruits CD4+ regulatory T cells (Tregs) that secrete suppressive cytokines (e.g., IL‐10 and TGF‐β), further driving T‐cell dysfunction or exhaustion.

#### 2.1.3 Immune Checkpoint Pathways

**FIGURE 2:**
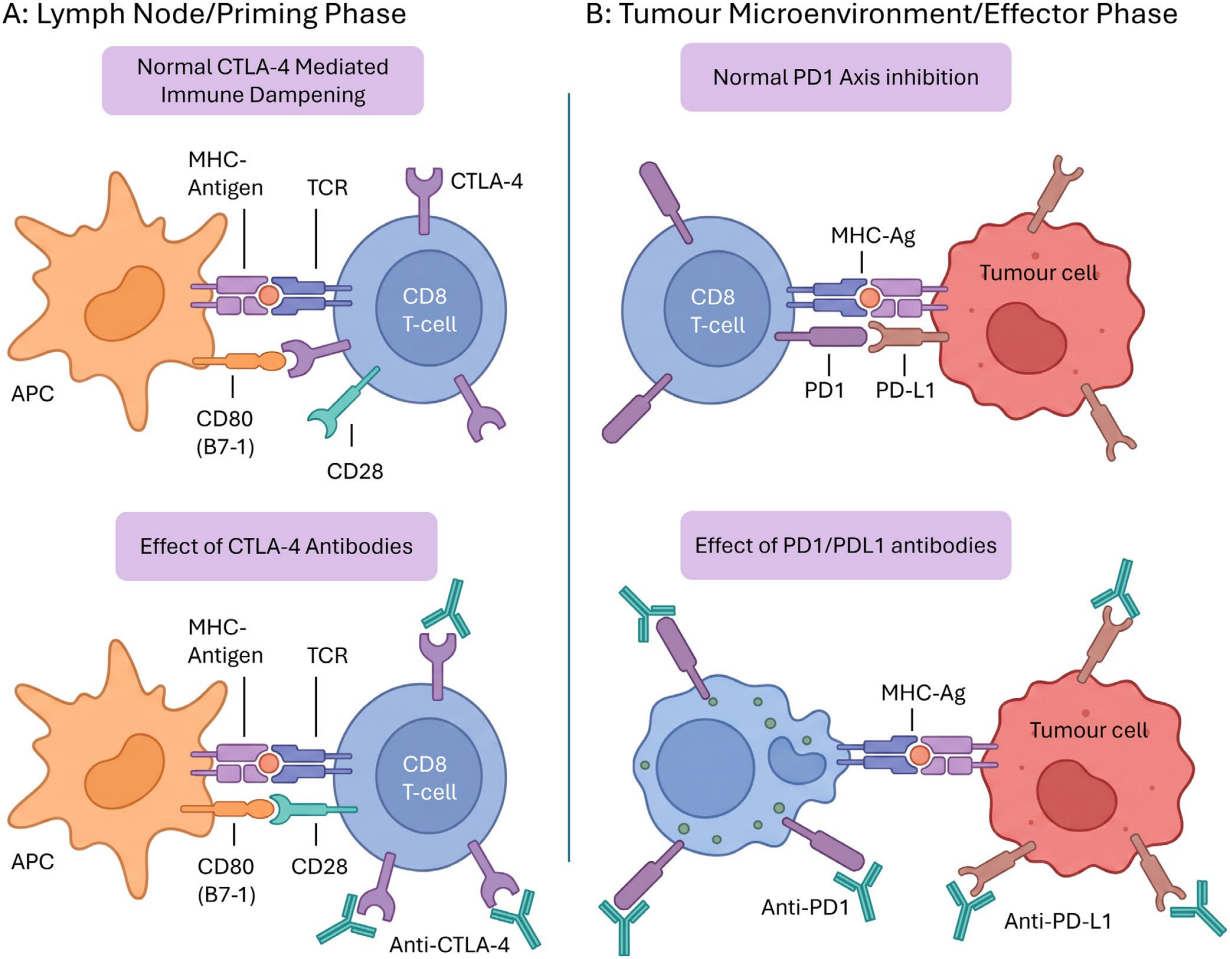
Mechanisms of CTLA‐4 and PD1 axis inhibition. A‐Top (Normal CTLA‐4 Mediated Immune Dampening): Following T‐cell activation, CTLA‐4 is upregulated on the T‐cell surface and competes with the costimulatory receptor CD28 for binding to the CD80/86 ligands on antigen‐presenting cells (APC). Because CTLA‐4 binds these ligands with significantly higher affinity than CD28, it effectively dampens the “go” signal, reducing proliferation and function of T cells. A‐Bottom (Effect of CTLA‐4 Antibodies): By blocking the CTLA‐4 receptor, anti‐CTLA‐4 antibodies prevent the inhibitory signal restoring the normal CD28 costimulatory signal to drive T‐cell priming, proliferation, and systemic anti‐tumor activity. B‐Top (Normal PD‐1 Axis Inhibition): PD‐1 is an inhibitory receptor expressed on T cells that have already been activated and have migrated to peripheral tissue. PD‐L1 expressed on the tumor cell binds to the PD‐1 receptor on the T cell, which transmits a negative signal that inhibits T cell proliferation and shuts down the release of cytotoxic granules. This interaction drives T cells into a state of exhaustion; they are present at the tumor site but rendered functionally inert. B‐Bottom (Effect of PD‐1/PD‐L1 Antibodies): Anti‐PD‐1/PD‐L1 therapies (e.g., pembrolizumab, nivolumab) competitively block this interaction directly within the tumor microenvironment, thereby reducing T‐cell exhaustion and reinvigorating exhausted T cells to release cytotoxic granules and eliminate malignant cells. MHC = major histocompatibility complex; TCR = T‐cell receptor (TCR). [Color figure can be viewed at wileyonlinelibrary.com]

Tumors co‐opt inhibitory checkpoints, which naturally preserve self‐tolerance, to effectively switch off tumor‐specific T‐cells. Immune checkpoint inhibitors (ICIs) restore cytotoxic potential by blocking these pathways at their two primary nodes (Figure 2).

CTLA‐4 (the central brake): Cytotoxic T‐lymphocyte‐associated protein 4 (CTLA‐4) operates centrally during the priming phase within lymph nodes to dampen initial T‐cell activation. Blockade allows for sustained T‐cell priming and the enhanced mobilization of new T‐cell clones against tumor antigens.

PD‐1/PD‐L1 (the peripheral brake): functions peripherally during the effector phase within the tumor microenvironment. Binding of PD‐1 to its ligands (PD‐L1 and PD‐L2) renders T‐cells functionally inert or exhausted [7]. Blocking either PD‐1 or PD‐L1 reinvigorates these cells to clear the tumor.

#### 2.1.4 Immunogenicity of Somatic Variation: TMB, Neoantigens, and Dynamic Monitoring

The immune system's ability to recognize and attack malignant cells is strongly correlated with the presence of immunogenic antigens expressed by the tumor. Many of these are “neoantigens,” proteins generated by somatic mutations that look foreign to the host. Tumor Mutational Burden (TMB) is defined as the total number of non‐inherited (somatic) mutations detected per megabase of interrogated genetic sequence, usually limited to the exome region and protein‐altering (nonsynonymous) variants [8]. Metastatic cutaneous SCC has the highest TMB reported at over 200 mutations per megabase [9]. It has emerged as a crucial predictive biomarker as high TMB correlates with an increased load of neoantigens, which serve as targets for T‐cell recognition. TMB is associated with improved response to ICI across different cancers including HNSCC [10] and retrospective analysis of biomarkers in 192 HNSCC patients in the KEYNOTE‐012 study confirmed an association between TMB and ICI response [11].

The American Society of Clinical Oncology (ASCO) guidelines recommend TMB testing may be performed when the PD‐L1 CPS is not available or in patients with rare tumors. Pembrolizumab may be offered as systemic treatment for TMB‐high (> 10 mut/Mb) recurrent or metastatic rare head and neck cancers [12].

Whilst high TMB alone provides valuable data for anticipating a therapeutic response, its utility as a standalone biomarker is limited due to substantial tumor heterogeneity [13], and studies have moved towards integrated molecular profiling for patient stratification, for example the LORIS score from Memorial Sloan Kettering Cancer Center which utilizes TMB, systemic therapy history, blood albumin level, blood neutrophil‐lymphocyte ratio (NLR), age and cancer type [10].

Beyond the single snapshot provided by pre‐treatment tissue biopsies, dynamic monitoring through liquid biopsy techniques offers potential for enhancing real‐time management and overcoming limitations posed by tumor heterogeneity. Liquid biopsy involves the non‐invasive sampling of body fluids, primarily blood or saliva, to detect biomarkers such as circulating tumor cells (CTCs), cell‐free tumor DNA (ctDNA), and viral DNA (vDNA). The use of these allows for ongoing real‐time assessment of tumor dynamics, providing early insights into treatment efficacy during the treatment course.

#### 2.1.5 T‐Cell Receptor (TCR) Repertoire Analysis

The analysis of the T‐cell receptor (TCR) repertoire which involves high‐throughput sequencing of the TCR β chain (TCRβ) complementarity‐determining region 3 (CDR3), aims to profile the diversity and clonality of circulating or tumor‐infiltrating T lymphocytes (TILs), reflecting the patient's immune response against malignancy. This approach has the potential to predict prognosis, and increased TCR diversity has been associated with improved response to ICI in head and neck cancer [14, 15]. Dynamic changes in the repertoire, specifically the expansion of dominant clones (increased clonality) during treatment, can serve as an early surrogate marker for therapeutic response [16].

#### 2.1.6 Molecular Architecture of Immune Escape and Rationale for Combination Therapies

Despite the success of immunotherapy, the potential to achieve durable objective responses (15%–20% in the recurrence/metastatic setting [17]) is limited by the multi‐layered mechanisms of immune escape employed by the tumor and its immune microenvironment (TIME). The TIME is a complex ecosystem that contributes to immunosuppression through the presence of dysfunctional or exhausted T cells, metabolic dysregulation (nutrient depletion and acidic pH), and recruitment and maintenance of suppressive immune cell populations.

Tumor cells secrete a variety of soluble factors to recruit cytotoxic‐dampening cells including regulatory CD4+ T cells (Tregs), myeloid‐derived suppressor cells (MDSCs), tumor‐associated macrophages (TAMs), and cancer‐associated fibroblasts (CAFs). Once present in the TME, these cells contribute to active suppression of anti‐tumor immunity. Tregs inhibit cytotoxic T cells [18], MDSCs deplete essential amino acids [19], and CAFs create a physical barrier and secrete immunosuppressive cytokines that induce a pro‐tumor M2 phenotype in macrophages [6].

The practical implication of this complex ecosystem is that relying on a therapeutic strategy that targets only a single immune checkpoint, such as the PD‐1/PD‐L1 axis, may be insufficient to mount an adequate immune response. Emerging therapeutic approaches are exploring specific nodes of resistance, for example combination regimens incorporating the IDO‐inhibitor BMS986205 [20], or the LAG‐3 inhibitor (relatlimab) [21] with nivolumab improved rates and degree of tumor response.

### 2.2 Mechanistic Limitations of Adjuvant Sequencing

There is a central conflict between traditional surgery and immunotherapy. For decades, our gold standard has been R0 resection followed by adjuvant therapy. However, biologically, this approach inadvertently creates a profound paradox.

Primary resection and lymph node dissection intrinsically depletes the biological substrates required for a robust immune response: the primary source of tumor neoantigens and the regional lymphoid structure required for T‐cell priming. This effectively disconnects the antigen‐presentation circuit, leading to insufficient antigenic drive to expand tumor‐specific clones.

Compounding this is the fact that the postoperative surgical bed harbors an intrinsically immunosuppressive microenvironment. Specifically, the physiological process of wound healing recruits a pro‐reparative cytokine milieu including TGF‐β and M2 macrophages, which actively suppress cytotoxic T‐lymphocyte activity. This results in a local immunosuppressive shield around potential minimal residual disease (MRD), which blunts the efficacy of adjuvant immunotherapy precisely where it is needed.

This mechanistic limitation is described as the post‐surgical “immune desert,” the immune system struggles to mount a robust response in the absence of both macroscopic disease and its regional lymphoid infrastructure. This biological constraint likely explains the failure of previous adjuvant monotherapy trials (e.g., IMvoke010 [22]), and the phase III JAVELIN Head and Neck 100 trial [23] which failed to demonstrate superior progression free survival with adjuvant immunotherapy and chemoradiation compared to standard chemoradiation.

Consequently, initiating systemic immunotherapy only after the extirpation of the primary tumor and regional lymphatics fundamentally limits its physiological efficacy.

### 2.3 Biological Advantages of Neoadjuvant Priming

This biological paradox provides the imperative for shifting therapy to the preoperative window. Unlike traditional induction chemotherapy, which historically failed to improve overall survival despite achieving radiographic volume reduction [24, 25], neoadjuvant immunotherapy facilitates systemic biological modulation.

#### 2.3.1 Rationale for Neoadjuvant Immunotherapy

By treating before surgery, we capitalize on the very immune substrates that surgery traditionally discards. The undisturbed primary tumor provides a high burden of neoantigens, and the intact lymphatic drainage ensures competent T‐cell trafficking and priming in regional lymph nodes. This process converts the tumor itself into an “in situ vaccine,” expanding a diverse T‐cell repertoire before the immune substrate is surgically removed. These expanded clonal T cells can then persist systemically to target micrometastatic disease long after the primary tumor has been resected.

This is supported by the phase III KEYNOTE‐689 trial which demonstrated that perioperative pembrolizumab significantly improved event‐free survival compared to standard surgery and adjuvant therapy alone [26]. By capitalizing on this intact immunologic substrate prior to resection, neoadjuvant approaches yield survival advantages not observed in purely adjuvant trial designs. Preclinical data suggest that the neoadjuvant component specifically is superior to adjuvant strategies for eradicating metastatic disease in oral cancer [27], and breast cancer [28] murine models.

A practical advantage lies in the clinical‐to‐pathologic downstaging observed in patients who achieve a Major Pathologic Response (MPR) or Pathologic Complete Response (pCR). This outcome offers a significant benefit to the surgeon by potentially enabling future function‐preserving, de‐intensified surgical approaches, pending the validation of these pathologic end points in long‐term survival studies. Reduction in the overall extent of surgical resection including de‐escalated surgical approaches aimed at preserving critical function (e.g., mandible or laryngeal preservation), and reduction in the need for complex free tissue transfer reconstruction [29].

#### 2.3.2 Promising Phase II Trials and Combination Approaches

Neoadjuvant ICI administration has demonstrated promising clinical activity in phase I and II trials, generally reporting favorable pathologic response rates and manageable safety profiles.

A systematic review of Phase I/II trials showed that immunotherapy alone (ICI monotherapy) achieved a pooled estimate for complete pathologic response of 1.4% [2], although another review reported pooled objective response rates (a less stringent measure of response) of 41.5% [30]. Crucially, trials demonstrated minimal toxicity with ICI monotherapy (pooled estimate of 8.5% for grade 3–4 adverse events) [2].

A favorable pathologic response to single‐agent ICI was observed even in HPV‐negative patients, a group with traditionally poor response to chemotherapy/radiotherapy. For example, neoadjuvant pembrolizumab yielded a pathologic response (≥ 50% tumor necrosis/giant cell reaction) in 22% of this challenging subgroup.

Importantly, a clear association has been established: patients who achieve a pathologic response following neoadjuvant pembrolizumab demonstrate significantly improved disease‐free survival compared to non‐responders [31].

More aggressive combination strategies have shown exceptional pathologic responses, albeit with increased toxicity.

- *Neoadjuvant chemoimmunotherapy*: combining ICI with chemotherapy results in a pooled cPR rate of 30.1%, significantly higher than monotherapy [2]. However, this benefit is associated with increased adverse events (22.9% grade 3+ AEs, potentially impacting surgical candidacy) compared to ICI alone.
- *Neoadjuvant immunoradiotherapy*: trials combining stereotactic body radiation therapy (SBRT) delivered to gross tumor volume with nivolumab have demonstrated remarkably high pathologic response rates. The Neoadjuvant Immuno‐Radiotherapy Trial (NIRT) reported a major pathologic response (< 10% residual viable tumor) rate of 86% with downstaging occurring in 90% of patients [32]. The pooled cPR rate for immunoradiotherapy was estimated at 54.7% [2]. Surgeons must balance this high efficacy against the elevated risk of local complications in the surgical field.

#### 2.3.3 Phase III Evidence

The compelling data from these earlier studies culminated in the phase III KEYNOTE‐689 trial (NCT03765918). This trial randomized patients with newly diagnosed, resectable LA HNSCC to receive perioperative pembrolizumab (two cycles neoadjuvant, 15 cycles adjuvant) plus standard care versus standard of care alone (surgery + risk‐adapted adjuvant (chemo)radiotherapy) [26].

While data confirmed that the addition of perioperative pembrolizumab significantly improved 36‐month event‐free survival (EFS) (57.6% vs. 46.4%), with the benefit most pronounced in PD‐L1 positive tumors (CPS ≥ 10), the clinical interpretation of these data warrants scrutiny [33]. A key limitation is that the trial restricted the definition of an EFS event to radiographic progression that precluded surgical resection. This definition inherently obscures cases where clinical or radiographic progression occurred during the neoadjuvant phase but did not ultimately abort the planned operative intervention. While this operation definition demonstrates that neoadjuvant immunotherapy did not significantly compromise the feasibility of surgical completion, granular data is missing. Specifically, the trial does not describe the required surgical footprint or subsequent treatment‐related morbidity among the 45 patients who progressed in the pembrolizumab cohort compared to the eight in the control arm.

While KEYNOTE‐689 established the utility of perioperative sequencing, NIVOPOST‐OP evaluated an exclusively postoperative paradigm [34]. This study demonstrated that the addition of concurrent and maintenance nivolumab to standard adjuvant cisplatin‐radiotherapy for patients with high risk resected locally advanced head and neck squamous cell carcinoma improved 3‐year disease‐free survival (63.1% vs. 52.5%; *p* = 0.034).

#### 2.3.4 HPV Status and Response

It is critical to note that while the majority of neoadjuvant data includes both HPV‐positive and HPV‐negative cohorts, the exceptional response rates often seen in studies are driven, in part, by the highly immunogenic nature of HPV‐positive disease. HPV‐positive HNSCC is biologically distinct, typically harboring a higher T cell infiltrate and carrying a generally better prognosis [35]. Crucially, however, favorable pathologic responses to single‐agent ICI have also been observed in HPV‐negative patients; neoadjuvant pembrolizumab yielded a pathologic response in 22% of this challenging subgroup [26]. This demonstrates that ICI can overcome the intrinsically colder immune microenvironment characteristic of many HPV‐negative HNSCCs.

### 2.4 Patient Selection: Biomarkers for the Surgical Oncologist

While TNM staging defines anatomical extent, it lacks predictive utility for immunotherapeutic response. Selecting candidates for neoadjuvant intervention requires moving beyond tumor size to evaluate the molecular landscape and the tumor immune microenvironment.

#### 2.4.1 Standard Biomarkers

Current clinical‐decision making relies on assessing whether an inhibitory target is present (i.e., PD‐L1) and if the tumor is immunologically foreign enough to be recognized (TMB/MSI) (Table 1).

**TABLE 1:** Biomarkers available for prediction and assessing immunotherapy response.

Biomarker	Source material	Predictive insight	Impact on surgical decision
PD‐L1 Expression (Combined Positive Score, CPS)	Assessed via IHC on tumor cells and immune cells, quantified by CPS	A higher CPS (≥ 1 or ≥ 20) correlates with superior ORR and OS benefit following anti‐PD‐1 therapy.	Most widely accepted predictive biomarker. Guides systemic therapy selection in recurrent/metastatic (R/M) settings (e.g., monotherapy vs. combination). In the neoadjuvant setting high CPS, alongside clinical or radiographic response, may support continuing ICI prior to definitive surgery
Tumor Mutational Burden (TMB)	Next‐generation Sequencing (NGS) or Whole Exome Sequencing (WES) on tissue samples. Defined as the number of somatic non‐inherited mutations per megabase	High TMB (≥ 10 mutations/Mb) is associated with an increased predicted neoantigen load, correlating positively with ORR to anti‐PD‐1 agents	TMB testing may be performed if the PD‐L1 CPS is unavailable [12], particularly in R/M or rare tumors, to identify potential responders to ICI. High TMB may support selection of neoadjuvant ICI regimens
Microsatellite Instability (MSI)	Assessed via genomic profiling; MSI‐High (MSI‐H) indicates defective DNA mismatch repair, indirectly assessed by absent DNA mismatch repair proteins on IHC	MSI‐H tumors are generally associated with a favorable response to anti‐PD‐1 ICIs. However, the incidence of MSI‐H HNSCC is reported to be relatively low (1%–3%) [36]	Standard MSI testing is generally recommended against in HNSCC due to low prevalence [12]. If confirmed, MSI‐H status provides a rationale for ICI treatment
Tumor‐Infiltrating Lymphocytes (TIL)/peripheral immune profiles	Evaluated via IHC (e.g., CD8+, FoxP3+) on tumor tissue, or via liquid biopsy methods like mass cytometry (CyTOF) and proteomics of peripheral blood mononuclear cells (PBMCs)	Increased infiltration of CD8+ TILs correlates with improved prognosis. Peripheral blood analysis can predict neoadjuvant response.	Analysis of pretreatment T cell subsets and inflammatory factors can aid in selection of patients most likely to benefit from neoadjuvant ICI
Gene Expression Profile (GEP) score	Typically refers to the T‐cell‐inflamed GEP (Tcell_inf_GEP), an 18‐gene signature characterized by IFN‐γ signaling, assessed via RNA sequencing	Independently associated with ORR and OS after pembrolizumab.	Offers complementary information to TMB and PD‐L1 [11]. High GEP score strengthens the rationale for selection for neoadjuvant regimens
Liquid biopsy (Circulating Tumor/Viral DNA (ctDNA/vDNA))	Analysis of circulating tumor DNA (ctDNA), circulating tumor cells (CTCs), or PD‐L1‐bearing exosomes from plasma or saliva	Dynamic monitoring of tumor burden clearance. ctDNA changes can precede radiographic progression allowing this to be identified early, clearance of ctDNA predicts excellent RFS	Neoadjuvant: offers a non‐invasive dynamic tool to monitor early treatment efficacy and distinguish pseudoprogression from true progression. Adjuvant/surveillance: ctDNA is sensitive for detecting minimal residual disease (MRD) post‐curative resection. Persistence of ctDNA suggests the need for adjuvant therapy intensification or closer follow‐up to manage impending relapse

Abbreviations: CPS = combined positive score; IHC = immunohistochemistry; ORR = objective response rate; OS = overall survival; RFS = recurrence free survival; R/M = recurrent or metastatic.

##### 2.4.1.1 PD‐L1 Expression

Programmed death ligand 1 (PD‐L1) expression remains the most widely accepted predictive biomarker. It is quantified using the Combined Positive Score (CPS); high expression (CPS of ≥ 1 or ≥ 20) correlates improved objective response rates [37, 38]. However it is an imperfect predictor; some low‐expressors still respond [39], meaning a low score does not strictly preclude neoadjuvant ICI.

##### 2.4.1.2 Tumor Mutational Burden (TMB)

Reflects the number of mutations per megabase of DNA. High TMB (≥ 10 mutations/Mb) suggests a higher neoantigenic load, making the tumor more visible to the immune system and correlates with improved responses across multiple cancers, including HNSCC [40].

##### 2.4.1.3 Microsatellite Instability (MSI)

Whilst MSI‐high (MSI‐H) tumors are exceptionally sensitive to immunotherapy and FDA approved for pembrolizumab in a tumor‐agnostic fashion, MSI‐H is rare in HNSCC, occurring in only 1%–3% of cases [36].

#### 2.4.2 Emerging Biomarkers and Dynamic Monitoring

Beyond static tissue biopsies, emerging biomarkers offer a more nuanced view of T‐cell functionality and real‐time tumor dynamics.

##### 2.4.2.1 Tumor‐Infiltrating Lymphocytes (TILs)

The presence of CD8+ T‐cells within the tumor stroma indicates a “hot” microenvironment and a primed host response. High density of these cells correlates with improved response to ICIs [2, 41]. Peripheral blood analysis assessing levels of central memory T‐cells (*T*_cm_) is also showing promise in this regard.

##### 2.4.2.2 Gene Expression Profiling (GEP)

The T‐cell‐inflamed GEP (Tcell_inf_GEP) is an 18‐gene profile of interferon‐gamma‐responsive activity. High scores predict better response [11] and correlate with improved overall survival regardless of treatment [42].

##### 2.4.2.3 Liquid Biopsy

This is perhaps the most transformative tool for the surgeon. By measuring circulating tumor DNA (ctDNA) or viral DNA (vDNA) from plasma or saliva, we can monitor treatment efficacy in real‐time [43, 44]. In the neoadjuvant setting, liquid biopsy can help distinguish pseudoprogression from true failure, clearing ctDNA levels signaling a response even when a CT scan shows a growing inflamed mass [45].

## 3 The Neoadjuvant Window: Diagnostic and Logistical Traps

### 3.1 Pseudoprogression: The Diagnostic Dilemma

The advent of ICIs has fundamentally altered the interpretation of oncologic imaging, introducing atypical response patterns that challenge conventional standards of care [46] (Table 2). Among these phenomena, pseudoprogression (PsPD) is critical for the surgical oncologist to understand. PsPD is defined as a transient increase in tumor burden or the appearance of a new lesion on imaging, followed by subsequent regression, without a change in therapy [47].

**TABLE 2:** Distinguishing immunotherapy response patterns: Impact on surgical triage.

Response pattern	Radiographic criteria (iRECIST/volumetric)	Unique HNSCC anatomical risk	Actionable surgical decision
Pseudoprogression (PsPD)	Initial findings meet RECIST PD criteria, designated as immune unconfirmed progressive disease (iUPD). Requires confirmatory scan (typically 4–8 weeks later) transient increase due to edema/inflammation.	High anatomical risk for tumors involving airway or major vessels, may demand rapid intervention regardless of cause	If clinically stable/improving: continue immunotherapy and obtain follow‐up imaging If clinically deteriorating requires immediate decision for intervention (e.g., surgical debulking, airway management, or transition to salvage therapy) Biopsy may be warranted for rapid confirmation
True Progression (PD)	Initial IUPD confirmed on subsequent scan. Progression is often confirmed by volume increase and clinical decline.	Unrestrained tumor growth carries risk for life threatening complications, including vascular blowout, nerve invasion and fistula formation.	Discontinue current ICI if symptomatic progression occurs. Transition urgently to effective salvage treatment (e.g., salvage surgery, chemotherapy, palliative radiation, or clinical trial)
Pathologic Complete/Major Response (pCR/MPR)	Disappearance of all target lesions. Pathologic complete response (pCR) defined as 0% residual viable tumor in the surgical specimen	Successful outcome, but requires careful tissue handling during resection due to residual fibrosis or inflammation. In cases of large tumors achieving CR near major vessels, complex reconstruction may still be necessary	Curative intent surgery is indicated to confirm pCR. Pathologic analysis (pCR/MPR) guides decision‐making regarding the subsequent need for adjuvant therapy

Abbreviations: ICI = immune checkpoint inhibitor; (i)RECIST = (immune‐related) response evaluation criteria in solid tumors; iUPD = immune unconfirmed progressive disease; pCR/MRP = pathologic complete response/major pathologic response; PD = true progression; PsPD = pseudoprogression.

#### 3.1.1 Mechanism and Incidence in HNSCC

Biologically, this radiological flare is not tumor growth, but is instead a manifestation of the activated anti‐tumor immune response. The phenomenon results from a massive infiltration of immune cells (including T and B lymphocytes), edema, necrosis, and inflammatory exudate within the tumor site, causing transient lesion enlargement [48]. While well‐documented in melanoma with an overall incidence of 3%–10% [48], true PsPD is rare in HNSCC, occurring in less than 2% of cases [49, 50].

#### 3.1.2 Anatomical Imperative and Diagnostic Challenge

However, this low incidence does not reduce clinical risk. The head and neck surgeon faces a unique “anatomical imperative.” Given the rigid anatomical confines of the visceral spaces of the neck, transient tumor enlargement risks acute airway compromise, neurovascular encasement, or compression. Consequently, expectant management of suspected pseudoprogression can carry significantly higher morbidity in HNSCC compared to other solid tumors.

Differentiation requires a deviation from standard Response Evaluation Criteria in Solid Tumors (RECIST 1.1) [51] criteria, which would erroneously label these patients as failures. In addition to immune‐RECIST (iRECIST) [52] principles, in HNSCC the decision to operate or wait depends on clinical stability [36].

- *Clinically stable*: if the scan is early in treatment and shows growth, but the patient is asymptomatic (i.e., no new pain, dysphagia) the patient has immune unconfirmed progressive disease (iUPD). This mandates continued therapy with a confirmatory scan in 4–8 weeks to definitively distinguish PsPD from true progression.
- *Clinically deteriorating*: in the presence of clinical deterioration such as evolving stridor, intractable pain, or incidental cranial neuropathy, functional decline supersedes radiological ambiguity and mandates immediate intervention.
- *The tie‐breaker*: in ambiguous cases where the airway is safe but the diagnosis is unclear, a biopsy can differentiate a tumor filled with viable cancer cells from one filled with necrotic debris and immune infiltrate (T and B lymphocytes, macrophages, and multinucleated giant cells).

### 3.2 Optimal Surgical Timing

Once the patient is initiated on immunotherapy, the critical question becomes the optimal timing of surgical intervention. The goal is to maximize the pathologic response without missing the window for resectability.

With historical induction chemotherapy protocols, long cycles failed to improve survival [24, 25]. Contemporary neoadjuvant immunotherapy regimens are optimized as brief interventions to maximize T‐cell priming without unduly delaying definitive resection [53]. Current trials generally schedule surgery within 4–6 weeks of the last dose [26, 32, 54, 55].

This window allows sufficient time for systemic T‐cell priming while avoiding risks associated with treatment delay, such as hyperprogression or functional compromise due to anatomical constraints. Recent data also suggests that extending treatment yields diminishing returns. For instance, a comparison of cemiplimab regimens showed that extending treatment from 6 to 12 weeks did not improve pathologic response rates (51% vs. 55%) [56].

An emerging strategy proposes a more flexible approach. The Response‐Adaptive Surgical Timing (RAST) protocol utilizes an early scan at 4 weeks to triage patients: non‐responders proceed to surgical resection to avoid delay, while responders are given an additional cycle to potentially deepen response. Whilst still being validated, it highlights a necessary shift away from rigid protocols towards decision‐making based on real‐time biology.

## 4 Execution: Operating in the Era of Immunotherapy

For patients receiving immunotherapy in the neoadjuvant setting, the operating room presents new variables that warrant a re‐evaluation of surgical planning, reconstruction, and complication management.

### 4.1 Feasibility and the Risk of Progression

A primary clinical concern with neoadjuvant protocols is the risk of disease progression or treatment‐limiting toxicity precluding definitive, curative intent resection. The data here is largely reassuring. ICI monotherapy is well‐tolerated, with grade 3 or higher adverse events (AEs) occurring at a pooled estimated incidence of 8.5% [2]. While the integration of chemoimmunotherapy increases the toxicity burden (22.9% grade 3+ AEs), the safety profile remains favorable when compared to traditional chemotherapy [57, 58], with the most common treatment‐related adverse events being cytopenias, fatigue and nausea [59, 60].

Most importantly, neoadjuvant ICI rarely delays surgery. Most trials report no unplanned surgical delays [32, 54, 55]. Even in the large KEYNOTE‐689 trial, surgical delays due to adverse events occurred in only 11/323 (3.5%) of patients [26]. Typically, only severe (grade 3) organ dysfunction or prolonged high‐dose corticosteroid requirements necessitate a delay. When delays do occur, they are generally secondary to manageable immune‐related effects, such as gastrointestinal toxicity, or chemotherapy‐induced complications like febrile neutropenia (0.7% in chemoimmunotherapy cohorts [2]).

### 4.2 The Hostile Field: Perception vs. Reality

There is a pervasive concern that immunotherapy induces a “hostile” surgical field, characterized by fibrosis and obliterated planes; a phenomenon well‐documented in thoracic surgery following neoadjuvant PD‐1 blockade [61].

#### 4.2.1 Intraoperative Findings

In the head and neck, this fear appears largely unfounded. Studies assessing neck dissection post‐immunotherapy generally report preserved tissue planes, and no instances of nerve sacrifices due to fibrosis [62]. Whilst the tissues may appear more hyperemic or inflamed, particularly after immunoradiotherapy, the dissection is technically feasible and generally uncomplicated [32].

#### 4.2.2 Wound Healing

Data regarding wound healing after neoadjuvant ICI is mixed but leans towards safety for monotherapy. Although some single‐arm trials have flagged delayed wound healing rates as high as 22.7% [54, 63], matched control studies suggest these rates are no different from treatment‐naive patients, implying that this rate reflects the baseline risk of complex head and neck surgery. Despite this reassurance, caution is required. When modalities are combined, the NIRT trial (immunotherapy and radiation) reported a 33% incidence of delayed wound healing [32].

While vigilance is required, current data with ICI monotherapy does not justify deviating from standard reconstructive paradigms. However, in cases involving preoperative immunoradiotherapy, the increased risk of delayed healing (33%) necessitates heightened surgical precaution, tension‐free closure, and a lower threshold for vascularized reconstruction. Free flaps remain the gold standard when required, though extra care with hemostasis in hyperemic fields is prudent.

#### 4.2.3 The Previously Irradiated Field

A distinct challenge arises in the salvage setting, where tissues are already fibrotic and poorly vascularized due to prior radiation. Adding ICIs to this environment may amplify specific high‐stakes risks. Emerging case reports have flagged an association between ICI and development of mandibular osteoradionecrosis (ORN) in previously radiated areas, potentially due to exacerbation of local inflammation within hypoxic, radiation‐damaged bone, tipping it into necrosis [64, 65]. In salvage cases involving immunotherapy, surgeons should maintain a lower threshold for suspecting ORN and may need to favor vascularized bone reconstruction over conservative measures.

### 4.3 Adjuvant Immunotherapy and Systemic Complications

Postoperatively, surgical management transitions to rigorous surveillance. Adjuvant immunotherapy typically begins 4–6 weeks post‐surgery [26, 66]. This phase is considered “safe” because the biological intervention occurs after the acute phase of wound repair is complete [64].

A significant postoperative consideration is the delayed onset of immune‐related adverse events (irAEs). These are not typical chemotherapy side effects; they are autoimmune phenomena that can affect virtually any organ system [36]. Common manifestations include dermatologic toxicities (e.g., rash), gastrointestinal pathology (e.g., colitis), and endocrinopathies [67]. Clear institutional protocols are required for recognizing and managing these potentially life‐threatening toxicities, which may require intervention with high‐dose glucocorticoids and potentially permanent cessation of the ICI agent [36].

Hypothyroidism is a frequent occurrence following anti‐PD‐1 treatment. For instance, in KEYNOTE 689, potentially immune‐mediated adverse events of any grade were reported in 43.2% of the ICI group, with hypothyroidism being the most common specific event (24.7% incidence) [26]. These events can occur months or even years after treatment cessation [68]. The surgeon must remain vigilant for non‐specific decline (e.g., fatigue or weight changes) that may signal a treatable endocrine deficiency rather than a true recurrence.

## 5 Defining Success: Interpreting Pathologic Response

The introduction of neoadjuvant ICI has rendered the traditional pathology report incomplete. Given the unreliability of radiological response (RECIST) in the neoadjuvant window, the resection specimen becomes the definitive measure of therapeutic efficacy. The surgeon must now look for specific quantitative and qualitative measures of success.

The pathologist's report should focus on two key quantitative metrics: Pathologic Complete Response (pCR), defined as the absence of any residual viable tumor (RVT) in the primary site and nodes, and Major Pathologic Response (MPR), defined as ≤ 10% RVT remaining. These are validated as surrogate endpoints that robustly correlate with improved overall survival [69].

Even in patients who do not achieve a complete response, the immune system leaves a distinct signature. Unlike the cell death seen with chemotherapy, immunotherapy response is characterized by “regression beds”; areas where the tumor has been replaced by fibrosis, dense inflammatory infiltrate, and keratinous debris. Specifically, the presence of tumor necrosis and giant cell/histiocytic reactions (reflecting macrophage activation) are positive prognostic indicators. The surgeon should actively seek documentation of these features. Even “partial” regression (< 50% viability) predicts significantly better outcomes compared to non‐responders [31, 70].

However, a significant challenge remains the lack of uniformity. Current trials often employ disparate grading scales and cut‐offs [30], making it difficult to compare response rates across different studies. Establishing expert consensus on scoring is an urgent next step for the field [71].

## 6 Future Directions: The Era of the Surgical Immunologist

The future of immunotherapy in head and neck cancer centers on surgical de‐escalation, leveraging exceptional neoadjuvant responses to reduce the “footprint” of resection and preserve critical function without compromising oncological safety.

### 6.1 Response Adapted Surgery

At present, for the vast majority of patients treated outside of a clinical trial, the safest approach remains resection of the original footprint. This conservative stance is justified by the risk of scattered microscopic residual disease and high cost of locoregional recurrence. However, evidence is building for a response‐adapted shift in surgical planning. Emerging evidence from the REMATCH‐2201 trial [72] indicates that in patients exhibiting a radiologic response ≥ 50% to neoadjuvant chemoimmunotherapy, narrowing surgical margins to 5–10 mm beyond the residual tumor boundary is oncologically feasible. Similarly, results from the neoCHANCE‐1 trial demonstrate that neoadjuvant immunotargeted therapy significantly increases minimal pathologic margins compared to primary resection controls, providing a biological “safety buffer” that may facilitate organ‐preserving resections in locally advanced hypopharyngeal and laryngeal cancers [73].

To assist the academic field with studies on response adapted surgery, Cracchiolo et al. have introduced a systematic classification system for surgery in oral cavity squamous cell carcinoma [74]. A surgical morbidity score indexing the extent of mucosal and soft tissue resection, skin sacrifice, bone preservation, surgical exposure, and reconstructive complexity is assigned to the proposed definitive operation at baseline versus the actual procedure executed post‐neoadjuvant therapy. By assigning a numerical score, studies can objectively measure the magnitude of surgical de‐escalation in neoadjuvant clinical trials.

The pursuit of function preservation hinges entirely on diagnostic confidence. De‐escalation cannot proceed without certainty of a robust response. While tissue markers like PD‐L1 are informative, they remain static. Future management relies on dynamic diagnostics. Circulating tumor DNA (ctDNA) holds immense potential for monitoring minimal residual disease (MRD). Clearance of ctDNA may serve as the green light for reduced surgical margins. Integration of gene expression profiles (GEP) and comprehensive immune profiling is expected to supersede single‐biomarker utility, enabling more accurate prediction of pathologic complete response preoperatively.

### 6.2 Overcoming Resistance

For tumors that do not respond to PD‐1 blockade alone, the therapeutic strategy is to dismantle the tumor microenvironment's resistance mechanisms. Future clinical algorithms will be driven by combination regimens (Dual ICI, ICI‐chemo). Novel checkpoint targets like LAG‐3 (NCT04080804 and NCT04326257), and TIGIT (NCT03708224 and NCT04665843) are being investigated to overcome T‐cell exhaustion. Other agents targeting metabolic dysregulation [75] or resistance nodes (e.g., IDO inhibitors [20]) are designed to convert immunologically “cold” tumors to “hot.”

### 6.3 Adoptive Cell Transfer and Tissue Procurement

Beyond immune checkpoint blockade, adoptive cell transfer (ACT) including tumor‐infiltrating lymphocyte (TIL) and gene‐engineered T‐cell receptor (TCR) therapy, represents an emerging frontier in head and neck oncology. While currently restricted to early‐phase clinical trials and with significant resource requirements, ACT offers a highly specific mechanism for targeting vital antigens (e.g., HPV E6/E7 and EBV) and cancer germline antigens such as MAGE‐A4 and KK‐LC‐1 [76]. Clinical activity has been demonstrated in metastatic, treatment‐refractory HNSCC, including cases resistant to PD‐1 inhibitors [77], and a trial is underway in the neoadjuvant setting (NCT04015336).

For the surgical oncologist, the primary technical consideration involves the procurement of high‐quality, viable tumor deposits required for ex vivo T‐cell expansion. Integrating these specialized harvest protocols into the surgical workflow will require precise coordination to ensure that tissue acquisition for cellular manufacturing does not compromise oncological margins or delay definitive resection.

### 6.4 Translational Integration

Concurrently, research must focus on optimizing the entire perioperative protocol through enhanced standardization and the development of effective combination regimens to overcome resistance mechanisms inherent to the tumor microenvironment (TME). Integrating immunotherapy into the management of resectable HNSCC requires the surgical immunologist to routinely interpret translational data such as ctDNA kinetics, T‐cell repertoires, and immune phenotypes to guide operative planning.

By integrating seamlessly within a multidisciplinary framework and actively utilizing the resection specimens as a research resource, the surgeon will remain the central figure in translating these molecular breakthroughs into survival and quality of life for our patients.

## Data Availability

Data sharing not applicable to this article as no datasets were generated or analysed during the current study.
